# A Retrospective Analysis of Pain Etiology in Middle-Aged Patients with Peripheral Neuropathy

**DOI:** 10.3390/medicina57080787

**Published:** 2021-07-31

**Authors:** Anna K. Szewczyk, Anna Jamroz-Wiśniewska, Konrad Rejdak

**Affiliations:** Department of Neurology, Medical University of Lublin, ul. Jaczewskiego 8, 20-954 Lublin, Poland; annajamrozwisniewska@umlub.pl (A.J.-W.); krejdak@yahoo.com (K.R.)

**Keywords:** peripheral neuropathy, polyneuropathy, neuropathic pain, epidemiology, laboratory markers, middle age, aging

## Abstract

*Background and Objectives*: Correct assessment and a multidisciplinary approach appear to be extremely important in preventing peripheral neuropathy and its complications. The purpose of this study was to find the correlations and dissimilarities between different types of peripheral neuropathy, the occurrence of pain, and laboratory results. *Materials and Methods*: This retrospective study assessed 124 patients who were hospitalized in our neurology department due to various types of sensory or motor disturbances. The patients were eventually diagnosed with peripheral neuropathy, based on the electrophysiological study, anamnesis, physical examination, and laboratory results. The whole group was subjected to statistical analysis. *Results*: The mean age of patients was over 56 years, with a slight woman predominance. A statistically significant (*p* < 0.05) relationship between the place of residence and gender was seen, where more men than women live in the rural area, while more women than men live in the urban area. Most often we observed symmetric, sensorimotor, demyelinating, inflammatory, and chronic neuropathy. More than 40% of patients reported pain. A statistically significant correlation between the evolution/severity and the occurrence of pain was seen in subacute type (*p* < 0.05) and small fibre neuropathy (*p* < 0.01). *Conclusions*: A higher incidence of peripheral neuropathy in middle-aged people will become essential in the aging society with lifestyle and chronic disorders. Peripheral neuropathy is slightly more common in women than men and its occurrence may be influenced by work performed or internal and external factors. In the study group, more than 40% of patients reported pain, therefore the pain measurement for each patient should be implemented and repeated at every visit. An assessment of sodium level and, in women, markers of neuroinflammation level in the various types of peripheral neuropathy may be an interesting direction for the future.

## 1. Introduction

Peripheral neuropathies include all conditions that involve damage to the peripheral nervous system (PNS), which is prone to various types of damage, including mechanical, toxic, and metabolic origin. The clinical symptoms of peripheral neuropathy depend on its severity (evolution), distribution (symmetrical or asymmetrical), affected the structure of the nerve cell (axonal, demyelinating, or mixed neuropathy) as well as the type of affected neurons (autonomic, sensory, or motor) [1,2]. In clinical practice, we can encounter multiple classifications of neuropathy, which intertwine with each other.

It is assumed that based on the duration of the symptoms, peripheral neuropathy may be divided into acute (diagnostic emergencies) and chronic (developing over several months) type [3] or acute, subacute, and chronic [4].

PNS is created by the somatic nervous system and autonomic nervous system, which allows us to distinguish in the classification different affected modalities: sensory, motor, autonomic, or mixed [5,6]. Different symptoms are present in the patient depending on which type of nerve fiber is the most damaged. In motor neuropathy, we can list symptoms such as progressive muscle weakness, fasciculations, or cramps [5]. Sensory neuropathy is characterized by positive (e.g., allodynia, hyperalgesia, pain, paraesthesia, dysesthesia, and hyperpathia) or negative signs (sensory loss, hypoesthesia, hypoalgesia, and reduced sweating) [7]. Autonomic damage can include symptoms such as cardiovascular symptoms, gastrointestinal disturbances, bladder and sexual dysfunctions, and sweating disturbances [8]. The standard elements that will lead to a correct diagnosis include clinical history and presentation, neurological examination (with the assessment of the involvement of fiber modalities and the distribution of symptoms), electrodiagnostic studies (nerve conduction studies (NCS) and needle electromyography (EMG)), laboratory testing and if necessary examination of the cerebrospinal fluid (CSF), imaging tests (nerve ultrasound or magnetic resonance imaging, additional examination e.g., computer tomography or positron emission tomography if necessary) or genetic testing [9]. The ultrasound technique enables imaging, measure and locate the nerve damage, and to estimate the severity of the injury. It may also help to evaluate intrinsic and extrinsic abnormalities, as well as to assess the muscles that can indirectly give information about nerve fibers. Therefore, it seems that ultrasound should be considered as one of the useful research tools not only in diagnostics but also in treatment (e.g., injection of specific drugs for anesthesia and pain relief) [10]. For the diagnosis of small fibre neuropathy (SFN) supplemented recommendations on the use of a skin biopsy with intraepidermal nerve fiber density (IENFD) appeared in 2010 [11], however, no clear diagnostic universal recommendations have been developed yet [12]. 

Disorders of toxic, metabolic, infectious, and hereditary etiology may affect the occurrence of damage in peripheral terminals of unmyelinated C, myelinated Aδ, and Aβ fibres or motor neurons, secondarily causing changes in their density and hyperexcitability [13,14]. The isolated damage to Aδ and C fibres has been widely described for SFN, while knowledge about this entity is constantly evolving [15,16]. In SFN damaged or destroyed nerves fibres are not visible in the NCS examination, therefore the disease is often underestimated or misdiagnosed for a long time. However, damage to the above-mentioned fibres is presented in the form of autonomic signs, incorrect temperature sensation, and pain [17,18]. Patients report varying degrees of severity of symptoms, among others: distal symmetric numbness, different types of pain (such as cold-like, pins and needles, burning, electric), loss of pinprick sensation with or without allodynia or hyperesthesia, but also autonomic features (e.g., dry mouth or eyes, cardiovascular or gastrointestinal disorders, erectile dysfunction) [12,19]. It seems that for the complete diagnosis confirmation is required (in addition to the skin biopsy): a normal result of NCS, clinical examination, and Quantitative sensory testing (QST), in comparison of findings with positive and negative signs [15,20].

Also, axonal damage (as a result of pressure or hypoxia) and its consequences, can lead to the fibres degradation and changes in channel expression and composition, which may subsequently provoke defective transmission and ectopic discharges [21].

The variety of symptoms and the multiplicity of etiology [22,23,24,25] make it difficult to estimate the overall prevalence of peripheral neuropathy [26]. Moreover, research shows that peripheral neuropathy goes hand in hand with morbidity, therefore, in the aging population, we can expect an increasing number of patients with symptoms of peripheral neuropathy [27]. Unfortunately, certain types of peripheral neuropathy are associated with a greater physical disability, poor health, and probably even higher mortality, also beyond its comorbidities [28]. Next to increasing age, a high hyperglycaemic burden understood as a high level of HbA1c and duration of the diabetes mellitus (DM), may lead to the manifestation of diabetic neuropathy (DN). Insulin resistance, hypertension, and different components of metabolic syndrome seen frequently in diabetes mellitus type 2 can be also considered as a risk factor. Studies indicate that more than 50% of patients with diabetes will develop diabetic distal symmetrical polyneuropathy (DSPN) and about one-fifth of them neuropathic pain as complications [29,30]. DSPN is one of the most common types of diabetic polyneuropathy, manifested in the form of “socks and gloves”. Less frequently are observed diffuse or focal neuropathies [30]. Metabolic causes of peripheral neuropathy are also associated with nutritional deficiencies such as cobalamin, copper, or vitamin E deficiency, encountered especially in the elderly and leading mostly to sensory axonopathies [31]. 

When considering the occurrence of neuropathic pain in the context of decreased productivity at work or absenteeism, its social impact and connection to quality of life can be clearly seen. The emergence of sleep disorders, depression, or anxiety are also observed [32,33,34]. Neuropathic pain (NP), appearing frequently in the course of neuropathy, in accordance with the International Association for the Study of Pain (IASP) is defined as “pain caused by a lesion or disease of the somatosensory nervous system”, while peripheral neuropathic pain as “pain caused by a lesion or disease of the peripheral somatosensory nervous system”. NP can be also classified as disease or clinical conditions associated with chronic pain [35]. Pain manifestation is similar regardless of the type of damage. The analysis of somatosensory abnormalities can be useful in differentiating: neuropathic and nociceptive pain [36,37]. 

Research conducted by Barbosa et al. [38], confirms that pain is a common symptom among the population aged 65 and older in Europe. Over half of the respondents reported self-reported pain (higher scores were obtained for spouse caregivers). Aging can lead to polypharmacy (and its adverse effects, hospitalizations, or iatrogenic illness), which is most often associated with multiple diseases. However, studies show that specific and invalidating symptoms, such as pain also contribute to increased drug use [39]. Furthermore, assessment of the effectiveness of analgesic treatment is limited as elderly people are often excluded from clinical trials. Sedation, increased fall rates, cognitive impairments, are listed among the side effects of drugs for the treatment of neuropathic pain, which may be risky for an elderly patient [40].

The aim of this study was to find distinctive clinical features between different types of peripheral neuropathy, the occurrence of pain, and laboratory markers.

## 2. Materials and Methods

This retrospective study included 124 patients, who were hospitalized in University Hospital in Lublin between 2019 and mid-2020 due to various types of sensory (e.g., hypoesthesia, hyperesthesia, paraesthesia, pain) or motor disturbances (cramps, muscle weakness). Patients’ anamnesis, physical examination (including neurological examination and pain assessment) as well as laboratory results were gathered from the subjects’ medical records. The patients were eventually diagnosed with polyneuropathy. The final diagnosis was based on an electrophysiological study including NCS and EMG, conducted by a trained neurophysiologist. Lumbar puncture and cerebrospinal-fluid (CSF) analysis were performed at the beginning of hospitalization and before administering medications. Protein level and cytosis were analysed. CSF protein was considered elevated when >45 mg/dL and cytosis > 15 cells/μL. Additionally, each patient had undergone an imaging examination (head or spine, depending on the presented symptoms). 

Taking into account parameters such as acuteness and time course of the clinical presentation but also comorbidities and laboratory results, we decided to divide our group into six types: acute (toxic and acute motor axonal neuropathy AMAN), subacute (e.g., diabetic, infectious, and idiopathic neuropathy), chronic (idiopathic and diabetic neuropathy), inflammatory (chronic inflammatory demyelinating polyneuropathy CIDP, Multifocal Motor Neuropathy MMN, Guillain–Barré syndrome GBS), hereditary (Charcot–Marie–Tooth disease CMT, Neurofibromatosis type I NF-1) and small fibre neuropathy. Furthermore, the group was categorised according to the type of nerve fibre involvement, distribution, and type of damage, the occurrence of pain was also taken into account. Afterwards, the obtained results were subjected to statistical calculations.

Statistical analysis was performed using Statistica software (version 13.3, StatSoft, Lublin, Poland). Data expressed on a quantitative scale was presented as mean with standard deviation (SD). Data expressed on a qualitative scale was presented as the number and percentage of the sample. The Chi-squared test (χ^2^) was used to compare the relationships between variables expressed in the qualitative scale. To evaluate the differences between the subgroups of patients the unpaired two-sample Student’s *t*-test, Mann-Whitney test, one way ANOVA (with Tukey post-hoc test), and Kruskal-Wallis test were used. One-dimensional logistic regression was used to assess the impact of the variables on the risk of pain. The level of statistical significance was set at *p* < 0.05.

To select proper literature for this study, a systematic literature search was prepared based on PRISMA guidelines, by using PubMed/MEDLINE and Pain journal databases. The studies were published in English. Titles and abstracts of publications were found using keywords, such as peripheral neuropathy, polyneuropathy, neuropathic pain, chronic neuropathic pain, pain, small fibre neuropathy, laboratory markers, or epidemiology.

## 3. Results

### 3.1. Demography

In total, 124 patients were included in the analyses (Table 1). We received a slight woman predominance: 50.81% (*n* = 63) females and 49.19% (*n* = 61) males. Their age ranged from 23 to 86 years old, the mean age was 56.69 ± 14.7, for women 56.00 ± 14.48, and for men 57.35 ± 14.99 (*p* > 0.05). As regards the place of residence, the majority of respondents 70.16% (*n* = 87) came from the urban area, in comparison with the rural area 29.84% (*n* = 37). Furthermore, a statistically significant relationship (*p* < 0.05) between the place of residence and gender was seen, where more men than women live in the countryside (64% vs. 35%), while more women than men live in the city (54% vs. 42%), which is related to the demographic structure of Poland.

Taking into account the distribution of the symptoms (*p* > 0.05), symmetrical manifestation was observed in 71.77% (*n* = 89), asymmetrical in 25.00% (*n* = 31), while asymmetrical with cranial nerve involvement—in 3.23% (*n* = 4) of responders.

Considering the type of the damage (*p* > 0.05), axonal injury was diagnosed in 24.78% (*n* = 28), demyelinating in 51.33% (*n* = 58) and the mixed (axonal and demyelinating injury) in 23.89% (*n* = 27).

Focusing on the occurrence of pain (Table 2), of 124 patients 41.13% (*n* = 51) experienced pain (*p* > 0.05). The mean age of patients reporting pain was similar to patients without pain (56.40 ± 13.14 vs. 57.10 ± 16.82, respectively). Pain was 1.16 times more common in women and more than two times more frequent in patients living in the city (*p* > 0.05).

There was a statistically significant correlation (*p* < 0.05) between the type of nerve involvement and the occurrence of pain, pain was reported in patients with sensorimotor and sensory neuropathy, while no patient suffering from motor neuropathy notified such symptoms. 

In regards regards to the distribution of symptoms, in patients with an asymmetric manifestation, the risk of pain was found to be 1.99 times higher than in patients with a symmetric distribution, while in patients with an asymmetric distribution and cranial involvement this ratio/chance was 1.19 times lower (*p* > 0.05).

Compared with axonal polyneuropathy, the risk of pain in patients with demyelinating and mixed polyneuropathy was lower by 2.44 times and 1.30 times respectively (*p* > 0.05).

Also, a statistically significant correlation between the evolution/severity and the occurrence of pain was seen (*p* < 0.001). In comparison with inflammatory type, the risk of pain was 3.03 times higher in subacute type (*p* < 0.05), 5.31 time higher in SFN (*p* < 0.01) and 1.06 time higher in chronic type (*p* > 0.05).

No associations were found between the age of diagnosis and the type of nerve fiber involved nor type of the damage (Table 3).

### 3.2. Laboratory Analysis

A statistical analysis of the laboratory tests results was prepared (Table 3) comparing the results obtained for type of fiber involvement and the type of damage. Data on gender and pain occurrence is presented in Table 4.

A statistically significantly higher level of sodium was demonstrated between motor and sensorimotor type of peripheral neuropathy (142.78 ± 1.90 and 141.36 ± 2.64 respectively), at Kruskal-Wallis test (*p* < 0.05). No statistical significance was obtained for other parameters, however, an abnormal fasting glucose level displayed for each group, except demyelinating type, draws attention (Table 3.).

It was demonstrated that the erythrocyte sedimentation rate (ESR) was higher in women compared to men (the mean 32.50 ± 28.47 vs. 12.28 ± 14.33), which is a statistically significant result (*p* < 0.001). The other parameters did not differ significantly between gender nor patients with and without pain (Table 4).

The lumbar puncture was performed for 61 patients. Among them, pain was reported by 21 patients, including 14 women and 7 men. The concentration of proteins in CSF was slightly higher for the group with pain 58.51 ± 85.54 vs. 57.42 ± 31.26 (*p* > 0.05). Differences in CSF cytosis levels can be observed between the two groups 5.00 ± 10.71 for the pain-free group vs. 2.98 ± 4.48 for the group with pain. Patients suffering from pain have higher fasting glucose levels and visibly lower levels of vitamin B12 as compared to the pain-free group 102.08 ± 18.85 vs. 100.51 ± 14.04 and 921.81 ± 2384.84 vs. 1414.50 ± 2184.85, respectively (*p* > 0.05).

## 4. Discussion

### 4.1. Epidemiology

Hanewinckel et al. [41] conducted a literature analysis to assess the prevalence and risk factors of peripheral neuropathy, based on the medical database. The results obtained vary considerably (e.g., due to different assessment protocol), however, low prevalence: 0.8–2.5 per 1000 applies (4–11% of cases was over the age of 50 years) to African and Middle Eastern countries, while 7.3–32.5 per 1000 people (about 30% of cases was over the age of 50 years) was observed in Europe. Averaging, in general population peripheral neuropathy prevalence ranges from 1% to 3%, for the elderly it is approximately 7% and depends on socioeconomic status, population structure and different risk factors. Also, studies carried out in the Netherlands evaluate disease prevalence at 5.5% in comparison to Dutch and the US population 4.0% and 3.9%, respectively [42]. Van Hecke et al. [43] the estimated prevalence of pain with neuropathic characteristics between 6.9% and 10% [44,45]. Studies carried out on the French population indicate that for chronic pain patients with neuropathic characteristics, 21.7% reported pain of any intensity and 25.6% reported intensity from moderate to severe. The prevalence of pain with a neuropathic component was higher in women (60.5%) and increased with age, having the greatest intensity between the fifth and sixth decades of life. Its prevalence was more frequent in rural areas than in large cities and up to 2 times more common in manual workers than managers [32]. Our analysis confirms that pain is more common in women between the ages of 50 s and 60 s, while most of them live in the city. A higher frequency of neuropathic pain in the middle-age population was also observed in a work carried out in Brazil [46].

### 4.2. Neuroinflammation

The appearance of chronic pain may be associated with a disruption of the physiological processes associated with the resolution of neuroinflammation, while in normal cases, inflammatory processes are protective, targeted, inhibitory, and controlled [47]. It happens that among one etiological group of peripheral neuropathy (e.g., diabetic) there occur patients with and without pain. Perhaps the profile of pain cytokines in patients with painful peripheral neuropathy may present the imbalance between anti-inflammatory and pro-inflammatory cytokines because of the genetic predispositions; similar disturbances were observed for chemokines. However, the inflammatory mediators can be influenced by life-style and comorbidities [48]. Surprisingly, higher scores of inflammatory biomarkers levels (WBC, CRP, and ESR) were noted in the painless group in comparison with the pain group. These findings do not support the hypothesis of a major role for enhanced inflammation in the pathogenesis of painful peripheral neuropathy, rather the development of painless peripheral neuropathy. Ziegler et al. obtained similar observations on the role of inflammation in diabetic painful neuropathy [49]. However, each group presented a borderline score of CRP and ESR.

Factors such as osteoporosis, diabetes mellitus, cardiovascular diseases, or even obesity may also influence the higher scores of inflammatory markers. For the female gender, changes in the endocrine system and natural processes of aging such as postmenopausal state, will be an additional factor (women evaluated in our study were around the age or postmenopausal) [50,51,52], which could have contributed to higher ESR scores.

### 4.3. CSF Markers

Analysis of the cerebrospinal fluid allows us to observe biochemical changes taking place in the central nervous system (CNS). It contains numerous molecules and cells, whose modification and concentration inform about changes taking place in the CNS. The protein composition, derived from blood-brain barrier (BBB) filtration and drainage of interstitial fluid from the CNS, also includes proteins involved in other, more general, mechanisms, such as proteolysis, inflammation, signaling, or protein binding. It appears that the majority of “pain proteins” (pain-related proteins in CSF) take part in the same processes, but the essential proteins responsible for pain signaling are present in CSF at low concentrations. Views on this subject remain divided and require further research [53,54]. 

Assessing the concentration of proteins in CSF, the mean concentration of proteins in motor, sensorimotor and demyelinating polyneuropathy exceeded the accepted norm. Also, too high concentrations of proteins were observed for the pain and pain-free group, but the first one presented higher scores (*p* > 0.05). Perhaps, this may be related to changes in the BBB permeability. The morphology and function of BBB can be influenced by a number of neurological diseases. An increase in the permeability of the BBB can lead to an influx of pro-inflammatory cytokines or immune cells into the CNS. It may be a response to long-term inflammation in the periphery or chronic pain [44]. The occurrence of inflammatory cells and the release of inflammatory mediators may lead to the activation of glial cells, while microglia are responsible for the immunity of the nervous system. Glial activation in CNS is also related to inflammatory injury and formation and maintenance of pathological pain, however, the role of glial cells in pain is not yet well understood [55,56].

### 4.4. Other Laboratory Markers

A relatively frequent problem of patients with peripheral neuropathy is vitamin B12 deficiency. Cobalamin deficits are also seen in chronic alcoholism, while taking medications (such as metformin or estrogen contraceptive pills) [57] but also in infections, inadequate dietary intake, or malabsorption [58]. Probably, the precise assessment of deficiencies should be assessed together with the assessment of its metabolites, i.e., methylmalonic acid and homocysteine [59]. 

The prevalence of cobalamin deficiency increases with age, it was observed that in people over 60 years of age, almost 6% suffer from this deficiency [60] and it is very common in elderly patients in neurology departments appearing in the form of neuropsychiatric and haematological complications [61,62]. This problem of shortages was seen in 2 patients (58 y woman and 60 y man) with subacute, peripheral neuropathy. Both presented macrocytic anaemia. First presented flaccid paresis, while the second had burning and pain in the lower limbs.

Interestingly, the group of women and the pain-free group showed very high levels of vitamin B12 (1369.31 ± 2819.98 vs. 1414.50 ± 2184.85), while the accepted norm in our laboratory is 211–911 pg/mL. The higher level of vitamin B12 in both groups may result from chronic pharmacological treatment, which could have been recommended by a family doctor before the patient was referred for further hospital diagnosis to prevent the onset of peripheral neuropathy.

According to the available literature, in pain, cobalamin supplementation helps to regenerate nerves, increases nerve conduction velocity, upregulates brain-derived neurotrophic factor, and decreases pain signaling by inactivating vanilloid receptors. B vitamins seem to reduce homocysteine levels and thus increase the production of neurotransmitters [63,64]. Methylcobalamin (activated form of vitamin B12) is also called “vitamin of painkiller” and categorized as a co-analgesic drug [65]. Significant effects of cobalamin (alone or as complementary therapy) were observed in diabetic neuropathy [66], postherpetic neuralgia [67], or alcohol-related neuropathy [68].

For preventive purposes, a quick and correct diagnosis of DN is fundamental. As prevention, change of lifestyle factors with weight loss, fall prevention, or foot care (in patients with DM) are mentioned [69]. Pain in diabetic polyneuropathy can be influenced by such factors as non-modifiable (age and sex) but also behavioural and social. In the second type body mass index and increased waist circumference are included. Diabetic comorbidities, such as hypertension and cardiovascular diseases seem to be simply coexisting factors but this is still uncertain [70]. According to Feldman et al., increased glucose levels may lead to nervous system dysfunction by increasing the production of reactive oxygen species (ROS) and ROS-mediated intracellular injury and cellular dysfunction. Also, increased glycolysis can promote neuronal injury by disruption of some metabolic pathways. Correct neuronal metabolism is preserved thanks to a properly operating mitochondrial membrane. Excess glucose and high fatty acid flux lead to decreased Adenosine triphosphate (ATP) production and a significant increase in production of ROSs leading to mitochondrial injury and secondary neuronal dysfunction. Cytokines and chemokines appearing in the background of oxidative/nitrosative stress lead to an increase in the inflammatory and immune response and intensify neuronal damage. Also, hyperlipidaemia appears to influence the occurrence of damage to the peripheral nervous system [71].

The above confirms an important role of glycemia and its proper control in preventing the appearance of peripheral neuropathy and its complications.

In our work, a statistically significantly higher level of sodium was demonstrated between motor and sensorimotor types of peripheral neuropathy. It seems that this may be an interesting direction for further research, however, it requires a larger group of respondents. In the available literature, we found no published studies on this topic. Meanwhile, we found many works whose authors lean on the role of voltage-gated sodium channels in peripheral neuropathy, specifically in the degeneration of axons, which can guide new therapeutic strategies in form of sodium channel blockers [72,73].

### 4.5. Limitations

Some limitations to the present study should be discussed. The occurrence of neuropathic pain will be associated with an increase in inflammatory parameters, however, it is difficult to unequivocally assess all obtained laboratory results due to many factors (comorbidities and the patient’s lifestyle) but also a small size of the study group. Also, a small sample does not allow for an accurate estimation of population-based prevalence rates, because of a small sample. The next step would be to validate the results in a broader clinical setting.

The analysis of patients was conducted retrospectively, for this reason, it was not possible to directly assess the physician’s decision-making and the implementation of all patients of the same laboratory test sets. Unfortunately, due to the inability to perform a required skin biopsy with IENFD, we could only suspect SFN and send the patient to a center capable of further evaluation.

## 5. Conclusions

Our study showed that peripheral neuropathy is slightly more common in women than in men. Assessing the place of residence, more affected women live in the urban area, whereas more men suffering from peripheral neuropathy inhabit the rural area. This difference may be related to the work performed and external or internal factors influencing the manifestation of the disease. The mean age of the patients was over 56 years, which indicates a higher incidence of the disease in the middle-age and older. Most often we observed symmetric, sensorimotor, demyelinating, inflammatory, and chronic peripheral neuropathy. The pain was reported in patients with sensorimotor and sensory neuropathy and in types, such as inflammatory, subacute, and small fiber neuropathy. Pain screening is essential, because more than 40% of patients reported it. The pain measurement for each patient should be implemented and repeated at each visit. The variety of symptoms, the multiplicity of etiologies, but also multiple, often overlapping classifications, pose another challenge in making the correct diagnosis.

There is a strong possibility that, in an aging society with lifestyle and chronic diseases affecting the occurrence of neuropathic problems, peripheral neuropathy will become one of the most important health problems all over the world. 

It seems that the assessment of sodium levels and, in women, markers of neuroinflammation in various types of peripheral neuropathy may be an interesting direction for further research.

## Figures and Tables

**Table 1:** Summary of demographic and clinical parameters.

Characteristic	Gender (Female) *n* = 63 (50.81%)	Gender (Male) *n* = 61 (49.19%)	*p* Value
Age of diagnosis, years	56.00 (±14.99)	57.35 (±14.48)	>0.05
Place of residence			
Village	13 (35.14%)	24 (64.86%)	**<0.05**
City	50 (54.47%)	37 (42.53%)	
Type of nerve fiber involvement *			
Sensory	0 (0.00%)	3 (100.00%)	>0.05
Motor	9 (50.00%)	9 (50.00%)	
Sensorimotor	48 (52.17%)	44 (47.83%)	
Distribution			
Symmetric	39 (43.82%)	50 (56.18%)	>0.05
Asymmetric	17 (54.84%)	14 (45.16%)	
Asymmetric with the involvement of the cranial nerves	1 (25.00%)	3 (75.00%)	
Type of the damage *			
Axonal	15 (53.57%)	13 (46.43%)	>0.05
Demyelinating	25 (43.10%)	33 (56.90%)	
Mixed	17 (62.96%)	10 (37.04%)	
Evolution/Severity			
Inflammatory	21 (50.00%)	21 (50.00%)	>0.05
Subacute	11 (40.74%)	16 (59.26%)	
Small fiber neuropathy	6 (54.55%)	5 (45.45%)	
Chronic	23 (57.50%)	17 (42.50%)	
Acute	1 (50.00%)	1 (50.00%)	
Hereditary	1 (50.00%)	1 (50.00%)	

Statistically significant differences are expressed in bold type (*p* < 0.05). * A group of 11 patients with suspected small fiber neuropathy (SFN) was excluded from the analysis because this diagnosis requires further confirmation.

**Table 2:** Summary of demographic and clinical parameters of patients with and without pain.

Characteristic	No Pain *n* = 73 (58.87%)	Pain *n* = 51 (41.13%)	*p* Value	Logistic Regression	
				OR (95% CI)	*p* Value
Gender					
Female	36 (57.14%)	27 (42.86%)	>0.05	1	
Male	37 (60.66%)	24 (39.34%)		0.86 (0.42–1.78)	>0.05
Place of residence					
Village	26 (70.27%)	11 (29.73%)	>0.05	1	
City	47 (54.02%)	40 (45.98%)		2.01 (0.88–4.62)	>0.05
Type of nerve fiber involvement *					
Sensorimotor	53 (57.61%)	39 (42.39%)	**<0.05**	1	
Sensory	1 (33.33%)	2 (66.67%)		NA	-
Motor	18 (100.00%)	0 (0.00%)		NA	-
Distribution					
Symmetric	53 (67.95%)	25 (32.05%)	>0.05	1	
Asymmetric	16 (51.61%)	15 (48.39%)		1.99 (0.84–4.69)	>0.05
Asymmetric with the involvement of cranial nerves	3 (75.00%)	1 (25.00%)		0.84 (0.26–2.72)	>0.05
Type of the damage *					
Axonal	14 (50.00%)	14 (50.00%)	>0.05	1	
Demyelinating	41 (70.69%)	17 (29.31%)		0.41 (0.16–1.07)	>0.05
Mixed	17 (62.96%)	10 (37.04%)		0.77 (0.44–1.33)	>0.05
Evolution/Severity					
Inflammatory	31 (73.81%)	11 (26.19%)	**<0.001**	1	
Subacute	13 (48.15%)	14 (51.85%)		3.03 (1.07–8.59)	**<0.05**
Small fiber Neuropathy	1 (9.09%)	10 (90.91%)		5.31 (1.75–16.11)	**<0.01**
Chronic	28 (70.00%)	12 (30.00%)		1.06 (0.77–1.48)	>0.05
Acute	0 (0.00%)	2 (100.00%)		-	-
Hereditary	0 (0.00%)	2 (100.00%)		-	-

NA—not applicable, CI—confidence interval, Values given: mean ± SD, statistically significant differences are expressed in bold type (*p* < 0.05). * A group of 11 patients with suspected small fiber neuropathy (SFN) was excluded from the analysis because this diagnosis requires further confirmation.

**Table 3:** Comparison of laboratory test results for the type of fiber involvement and the type of damage.

Characteristic	Sensory Type *n* = 3 (2.65%)	Motor Type *n* = 18 (15.93%)	Sensorimotor Type *n* = 92 (81.42%)	*p* Value	Axonal *n* = 28 (24.78%)	Demyelinating *n* = 58 (51.33%)	Mixed *n* = 27 (23.89%)	*p* Value
Age of diagnosis	51.33 (±17.67)	52.61 (±12.14)	59.17 (±13.83)	>0.05	58.25 (±12.08)	57.50 (±14.30)	58.48 (±14.81)	>0.05
WBC × 1000/μL	5.65 (±3.63)	6.25 (±1.89)	6.49 (±2.06)	>0.05	6.19 (±2.32)	6.49 (±2.03)	6.54 (±1.88)	>0.05
CRP mg/L	-	8.04 (±17.13)	9.63 (±21.38)	>0.05	12.35 (±27.08)	8.64 (±14.46)	8.03 (±24.73)	>0.05
ESR	-	11.44 (±10.33)	22.42 (±25.37)	>0.05	23.21 (±28.85)	18.93 (±20.16)	22.38 (±24.86)	>0.05
Vit. B12 pg/mL	-	503.67 (±285.20)	1262.34 (±2588.59)	>0.05	1079.46 (±2071.99)	1332.57 (±2832.33)	523.90 (±270.46)	>0.05
Potassium (K^+^) mmol/L	4.83 (±0.29)	4.36 (±0.42)	4.30 (±0.40)	>0.05	4.27 (±0.42)	4.33 (±0.36)	4.36 (±0.49)	>0.05
Sodium (Na^+^) mmol/L	140.67 (±3.06)	142.78 (±1.90) *	141.36 (±2.64) *	**<0.05**	142.26 (±2.31)	141.53 (±2.54)	140.96 (±2.84)	>0.05
Glucose mg/dL	113.67 (±20.21)	103.86 (±11.99)	100.91 (±18.30)	>0.05	110.92 (±27.13)	98.10 (±10.40)	100.08 (±14.77)	>0.05
Protein in CSF mg/dL	-	81.60 (±119.00)	55.14 (±59.56)	>0.05	40.39 (±14.75)	75.18 (±93.07)	38.60 (±15.34)	>0.05
Cytosis in CSF cells/μL	-	2.20 (±1.69)	4.16 (±8.54)	>0.05	2.92 (±3.30)	5.00 (±10.20)	1.93 (±1.58)	>0.05

* Statistically significant difference at *p* < 0.05 Kruskal-Wallis test, statistically significant differences are expressed in bold type (*p* < 0.05). Values given: mean ± SD. WBC—white blood cells, CRP—C reactive protein, ESR—erythrocyte sedimentation rate, Vit. B12—vitamin B12, CSF—cerebro-spinal fluid.

**Table 4:** Comparison of laboratory test results of patients with and without pain.

Characteristic	Gender (Female) *n* = 63 (50.81%)	Gender (Male) *n* = 61 (49.19%)	*p* Value	No Pain *n* = 73 (58.87%)	Pain *n* = 51 (41.13%)	*p* Value
Age of diagnosis	56.00 (±14.99)	57.35 (±14.48)	>0.05	57.10 (±16.82)	56.40 (±13.14)	>0.05
WBC × 1000/μL	6.34 (±2.00)	6.65 (±2.12)	>0.05	6.64 (±2.06)	6.38 (±2.07)	>0.05
CRP mg/L	11.26 (±24.55)	7.21 (±11.78)	>0.05	12.12 (±20.91)	7.70 (±19.55)	>0.05
ESR	32.50 (±28.47)	12.28 (±14.33)	**<0.001**	21.59 (±22.80)	19.31 (±23.58)	>0.05
Vit. B12 pg/mL	1369.31 (±2819.98)	877.46 (±1519.454)	>0.05	1414.50 (±2184.85)	921.81 (±2384.84)	>0.05
Potassium (K^+^) mmol/L	4.34 (±0.44)	4.31 (±0.38)	>0.05	4.30 (±0.46)	4.34 (±0.37)	>0.05
Sodium (Na^+^) mmol/L	141.68 (±2.71)	141.53 (±2.43)	>0.05	141.38 (±2.47)	141.77 (±2.63)	>0.05
Glucose mg/dL	102.38 (±19.60)	100.29 (±13.22)	>0.05	100.51 (±14.04)	102.08 (±18.85)	>0.05
Protein in CSF mg/dL	52.42 (±67.66)	64.68 (±71.74)	>0.05	57.42 (±31.26)	58.51 (±85.54)	>0.05
Cytosis in CSF cells/μL	2.92 (±4.71)	4.74 (±9.84)	>0.05	5.00 (±10.71)	2.98 (±4.48)	>0.05

Values given: mean ± SD, statistically significant differences are expressed in bold type (*p* < 0.05). WBC—white blood cells, CRP—C reactive protein, ESR—erythrocyte sedimentation rate, Vit. B12—vitamin B12, CSF—cerebro-spinal fluid.
